# American Veterinary College

**Published:** 1887-04

**Authors:** 


### AMERICAN VETERINARY COLLEGE.

The twelfth Commencement exercises of the American Veterinary College took place on the 4th of March, at Chickering Hall, before the usual large gathering of friends of the institution. The following gentlemen received their degree of D. V. S. at the hands of the President of the Board of Trustees, as they were called by Professor A. Liautard, Dean of the Faculty:

T. W. Apeldorn, of Pa.
Don C. Ayer, of Kansas.
Leidy J. Backman, of Pa.
Roscoe R. Bell, of N. Y.
George C. Blaker, of N. J.
Daniel S. Breslin, of N. Y.
Andrew Hyde, of Ct.
Joseph C. Jackson, of N. Y.
James L. Kidd, of Ky.
Patrick F. Kiernan, of Mo.
Frederick Lamberton, of Mass.
John F. McGrath, of R. I.

George Bridges, of Ct.
Solomon Bock, of Wy. Ter.
Ephraim Booth, of N. Y.
Richard E. Buckley, of N. Y.
Henry Cady, of N. Y.
Andrew G. Collins, of N. Y.
Willis W. Curry, of N. Y.
John D. Deronde, of N. Y.
Henry H. Dews, of Mass.
John D. Fair, of Ohio.
William H. Fairbanks, of Mass.
Joseph R. Hammond, of N. Y.
Wilfred F. Harrison, of N. J.
John D. Hartman, of Pa.
William H. Hitchings, of N. Y.
Julius Huelson, Jr., of N. J.
William T. Miller, of Pa.
John S. Meyer, of Mo.
Oliver R. Moyer, of Pa.
Herbert Neher, of N. Y.
William H. Nice, of Pa.
William S. Ortgies, of N. Y.
Albert T. Sellers, of Pa.
John J. Shoemaker, of Ind.
Wilder D. Southwick, of Mass.
Oliver H. Tims, of N. Y.
William J. Tomlison, of Pa.
Richard L. Tucker, of West Ind.
Thomas J. Turner, of Mo.
Lewis C. Wakefield, of Vt.
George W. Werner, of Ill.
Joseph Shua, of N. Y.

The following prizes were granted as follows:

Dr. Julius Huelson, Jr., received the gold medal of the Board of Trustees for the best general examination.

Dr. Roscoe R. Bell received the set of veterinary and medical books, given by the Alumni Association, for the second best general examination.

Dr. James L. Kidd received the gold medal of the Board of Faculty for the best practical examination before a committee of veterinarians appointed outside of the Faculty.

Dr. J. D. Fair received the silver medal of the Michener prize for the best written and defended paper presented before the Class Faculty.

